# A survey-based cross-sectional study of doctors’ expectations and experiences of non-technical skills for Out of Hours work

**DOI:** 10.1136/bmjopen-2014-006102

**Published:** 2015-02-14

**Authors:** Michael Brown, Dominick Shaw, Sarah Sharples, Ivan Le Jeune, John Blakey

**Affiliations:** 1 Human Factors Research Group/Horizon Digital Economy Research, University of Nottingham, Nottingham, Nottinghamshire, UK; 2 Department of Respiratory Medicine, University of Nottingham, Nottingham, Nottinghamshire, UK; 3 East Midlands Academic Health Science Network, Nottingham, Nottinghamshire, UK; 4 Department of Clinical Sciences, Liverpool School of Tropical Medicine, Nottingham, Nottinghamshire, UK

## Abstract

**Objectives:**

The skill set required for junior doctors to work efficiently and safely Out of Hours (OoH) in hospitals has not been established. This is despite the OoH period representing 75% of the year and it being the time of highest mortality. We set out to explore the expectations of medical students and experiences of junior doctors of the non-technical skills needed to work OoH.

**Design:**

Survey-based cross-sectional study informed by focus groups.

**Setting:**

Online survey with participants from five large teaching hospitals across the UK.

**Participants:**

300 Medical Students and Doctors

**Outcome measure:**

Participants ranked the importance of non-technical skills, as identified by literature review and focus groups, needed for OoH care.

**Results:**

The focus groups revealed a total of eight non-technical skills deemed to be important. In the survey ‘Task Prioritisation’ (mean rank 1.617) was consistently identified as the most important non-technical skill. Stage of training affected the ranking of skills, with significant differences for ‘Communication with Senior Doctors’, ‘Dealing with Clinical Isolation’, ‘Task Prioritisation’ and ‘Communication with Patients’. Importantly, there was a significant discrepancy between the medical student expectations and experiences of doctors undertaking work.

**Conclusions:**

Our findings suggest that medical staff particularly value task prioritisation skills; however, these are not routinely taught in medical schools. The discrepancy between expectations of students and experience of doctors reinforces the idea that there is a gap in training. Doctors of different grades place different importance on specific non-technical skills with implications for postgraduate training. There is a pressing need for medical schools and deaneries to review non-technical training to include more than communication skills.

**Strengths and limitations of this study**

- This study explores a vital but under-researched area of medical care, drawing conclusions with practical consequences.
- The consistent effects and statistical significance of effects after a conservative correction for multiple testing suggests the relatively small sample size was sufficient to identify a true effect.
- The limited time frame (1 month) of this cross-sectional study ensures clear boundaries between levels of experience into which participants were grouped.
- The sample itself was biased both geographically (the UK only) and contextually (teaching hospitals), limiting the generalisability of the findings.
- As purely a self-report, the study could have been influenced by responder bias.

## Background

Hospitals in the UK are struggling to cope with the demands of ever increasing numbers of admissions. The past decade has seen a 37% rise in emergency admissions^1^ and falling bed numbers.^2^ These admissions are predominantly for the elderly and increasingly for individuals with multiple medical problems.^3^ The pressure of these changes is felt most keenly during the 75% of the year that does not fall between 9:00 and 17:00 from Monday to Friday. During this ‘Out of Hours’ (OoH) period, care is largely provided by a skeleton staff of junior doctors, support workers and nurses.^4^

### Junior doctors under pressure

The small and relatively junior OoH team must provide timely care for emergency admissions and acutely unwell patients on the wards. Over the past decade, lengths of stay have fallen, multimorbid patients have become more common^5^ and an increasingly complex range of diagnostic test and treatments are delivered routinely and with minimal delay (eg, a UK-wide primary coronary angioplasty). These changes have shifted a high volume of medical activity into the evenings, nights and weekends. Over the same time period, individual junior doctors’ working hours have fallen by more than 35% to comply with the European Working Time Directive.^6^ These factors have contributed to increased shift intensity and cross-specialty cover, and to problems of communication between and across shifts.^1^

These challenges are magnified by the increased stress, decreased support and potential fatigue when working OoH. Failure to perform well may delay patient care and impact on its quality, with well-documented effects on error rate and adverse outcomes,^7^ ^8^ and there is growing evidence that this high demand role leads to emotional burnout and a worse quality of life.^9^

Jackson and Moreton^10^ explored junior doctors’ awareness and use of best practice for night shift work and found that knowledge of issues, such as the effects of sleep inertia on alertness, was very low. They also reported that feeling lethargic or unwell during night shifts was common. Many of their respondents also reported instances of not being able to take breaks or rest during night shifts. Objective assessment of these aspects of shift work is increasingly possible from routinely collected data and such observations suggest a need for increased training in non-technical skills.^11^ Such studies are providing increasing evidence to support the existing concerns over the preparedness of new junior doctors,^12^ which appears to relate to the significant increase in mortality seen in August when new doctors start work.^13^

### Medical school training in non-technical skills

The vast majority of Medical School training focuses on teaching facts and technical skills. This approach is aimed at reducing errors relating to lack of knowledge. Although patient communication skills are now formally taught, little emphasis is given to other non-technical skills such as task prioritisation, handover, managing personal needs and communication with other staff. Situational Judgement Tests (SJT) involve presenting students with a work-related scenario and asking how they would respond.^14^ They were introduced across UK medical schools in 2012 and form 50% of the total application score that determines whether a medical student gains their junior doctor post of choice. Although SJTs have the potential to deliver a valid assessment of performance, they reflect the curriculum and thus tend to provide an assessment of best practice knowledge and are ineffective at differentiating between candidates’ work-related performance.^15^ Specifically, they do not address the range of non-technical skills required to function during OoH work.

Medical students are also less likely to acquire such skills through apprenticeship and mentoring than previous generations: revisions to medical teaching have been necessary to cope with the doubling of medical student numbers in the UK between 1998 and 2004.^16^ This trend has resulted in fewer hours spent on the wards and more time spent with ‘educational facilitators’ who have limited medical training.^17^ Both research and media attention in recent years have highlighted the importance of considering human factors in the training of clinical personnel.^18^ ^19^ Much of this work focuses on team working, but other issues like the management of personal needs and task prioritisation need more attention.^20^ ^21^

The following section describes a study in which we explore attitudes towards the non-technical skills necessary for OoH clinical working. Our aim was to establish which non-technical skills were most important, and if expectations of medical students differed from experiences of working doctors.

## Focus groups

In order to identify the key non-technical skills requirement for OoH care, a literature review and short series of focus groups with junior doctors and medical students were performed exploring their expectations and experiences of OoH work. Small focus groups were used as it was felt that larger focus groups would not have given individuals time to explore their experiences in detail while interviews would lack the validation and embellishment of ideas provided by group discussion.

### Method

We ran three focus groups, each 1 h long, with a total of six junior doctors and one final year medical student. These were run in a semistructured manner with the researcher following prompts exploring three general topic areas: general expectations, general experiences and non-technical skills.

The recordings were transcribed and content analysis was performed in order to identify common themes in terms of non-technical skills for OoH care.

### Results

Eight skills were consistently identified as important across the groups.

#### Managing sleep

Managing sleep was mentioned as a worry during medical school, but not experienced as being particularly problematic once working OoH care.

I was worried about lack of sleep. (Participant 1)

Sleep cycles much easier to get into than I expected. I was able to work well at 4am. (Participant 4)

#### Managing personal needs

Conversely, managing personal needs such as toilet breaks and eating enough was something that most participants did not realise would be an issue, but found problematic once working OoHs.

No time for toilet break etc. (Participant 3)

Initially only go for a break when you've done every job. But you soon realise that there isn't a time when you get a lull, you have to make time for yourself. (Participant 2)

This issue is tied to task prioritisation, but also involves understanding the value of prioritising personal needs and weighing longer term risks/benefits.

#### Dealing with clinical isolation

Having to care for patients in relative isolation was a major concern for many of the participants before and during their first OoH shifts.

Worried about being with someone who is really ill and not supported, unable to contact the registrar. (Participant 7)

#### Communication with senior doctors

This skill is closely linked with ‘Dealing with Clinical Isolation’ as establishing appropriate lines of communication with senior doctors is a key part of avoiding clinical isolation and dealing with complex situations effectively.

I was afraid to wake up consultants for minor issues. (Participant 4)

#### Route planning

Many participants mentioned getting lost and/or using route knowledge to optimise their OoH work. They reported a huge variation in the importance of route optimisation depending on site and found that when working on large sites it was a vital part of task prioritisation, but simply not an issue when only covering a few wards. They felt it was most important during crash calls, where time is of the essence and you may be called to a ward that you have never been to before. Nearly half of the participants reported the incidence of getting lost on the way to a crash call.

Crash calls are often slowed when you don't know where it is. Ward names can give no indication of where they are. Even registrars get lost some times. (Participant 6)

Lots of experiences getting lost, need to ask nurses or hope you bump into someone else on a crash call. Even when you know where to go, you often don't go the best way. (Participant 3)

#### Task prioritisation

A consistent theme throughout the sessions was how proper task prioritisation is vital for OoH working. When prompted to discuss task management, there was a clear feeling that it is a skill that you constantly develop throughout your medical career, taking years to master.

The biggest thing is the ability to prioritise. What needs doing right now, in a few hours or left to the next shift. (Participant 1)

#### Communication with nurses

Several participants mentioned the importance of communicating with nurses, mainly focusing on how to say no to their non-urgent request and asking them for help when appropriate.

It is important to be able to say no in a nice way, rather than trying to do everything someone asks you to do. (Participant 3)

#### Communication with patients

It was clear from the sessions that all participants had an assumption that communication with patients is important, as they have been told this throughout medical training. This particular skill was usually mentioned as a benchmark of importance against which other skills are compared.

Communication with staff, learning to say no to non-urgent task. Learned with lots of experience. Just as important as communicating with patients. (Participant 6)

## Survey

We used these initial results to develop a survey exploring junior doctors’ experiences and medical students’ expectations of night shift work, focusing on non-technical skills.

### Method

An online survey was developed using Qualtrics (http://www.qualtrics.com), consisting of three parts. The first section asked participants to state their stage of medical training. A second section of the survey asked them to rank the importance of the eight non-technical skills identified in study one, in order of importance for effective OoH shift work, for which the section of tied ranks was not allowed.

The survey was distributed via a weblink emailed to junior doctors and medical students in five NHS trust hospitals in England: Queens Medical Centre (Nottingham), Nottingham City Hospital, Glenfield Hospital (Leicester), Leicester General Hospital and Leicester Royal Infirmary. Responses were collected over the course of a month in spring 2013.

As ordinal data, rank-based methods are used for all inferential statistics with a Bonferroni correction for multiple tests applied as appropriate (see online supplementary table S3 for details). A Friedman test was used to determine the effect of skill on rank assignment. Post Hoc Stepwise Stepdown Multiple Comparisons were used to identify subsets of tasks with homogeneous characteristics, as described by Campbell and Skillings.^22^ Kruskal–Wallis tests were used to investigate the effect of stage in clinical education on the ranking of skills. The investigations were undertaken in SPSS (IBM, USA).

## Results

### Participants

A total of 480 potential participants were sent the survey, of whom 380 responded to the survey. Subsequently, 76 of these responses were removed prior to analysis due to incomplete responses and a further four doctors’ responses were removed as they had not yet experienced OoH work. See table 1 for a breakdown of the remaining 300 participants by level of experience.

**Table 1:** Breakdown of study 2 participants by level of experience

Group	Grade(s)	Approximate years of experience	Number of participants
0—Medical students	Student	0	66
1—New junior doctors	F1	<1	32
2—Junior doctors	F2, CT1 and CT2	1–3	89
3—Experienced junior doctors	ST3-ST9	4+	113

### Ranking of non-technical skill importance

**Figure 1:**
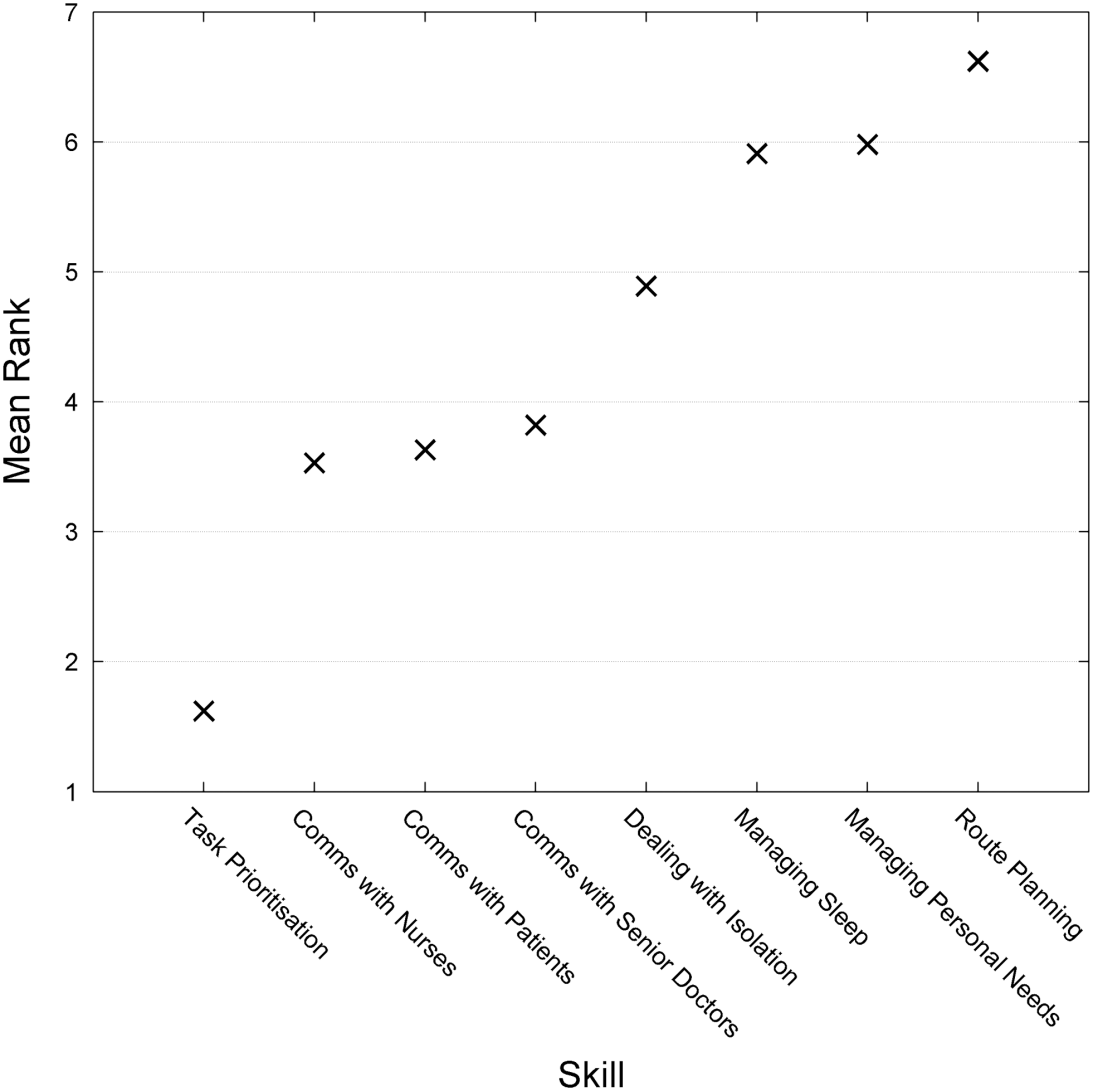
Mean rank of non-technical skills. 1=most important, 8=least.

There was a consistent difference in the way skills were ranked (χ^2^=966, df=7, p<0.001). Task prioritiation was felt to be most important, followed by communication skills (see figure 1). Communication with patients and with nurses were ranked equally (p=1.000) as were managing sleep and managing personal needs (p=0.235).

**Figure 2:**
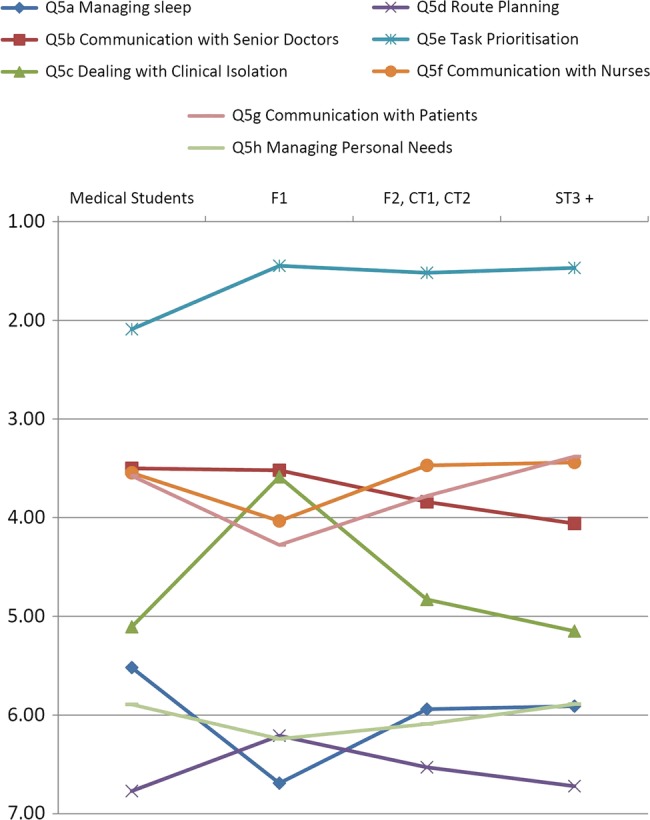
Mean rank of non-technical skills for Out of Hours care by stage of training.

Stage of medical training was associated with differences in ranking patterns for Task Prioritisation (p=0.002), Dealing with Clinical Isolation (p=0.006), Communication with Senior Doctors (p=0.024), and Communication with Patients (p=0.038). Medical students ranked task prioritisation significantly lower than did doctors (p=0.029 vs F1, 0.002 vs F2/CMT and 0.001 vs ST3+). Newly qualified doctors ranked dealing with clinical isolation higher than did the other doctors (p=0.005 vs F2/CT and p=0.001 vs ST3+). More experienced doctors ranked Communication with Patients higher than F1 doctors (p=0.038) and F2/CT doctors (p=0.009), but ranked ‘Communication with Senior Doctors’ as less important than medical students (p=0.004) or F1s (p=0.049). These differences are illustrated in figure 2, in which mean ranks presented as median/mode graphs were found to obscure some of the subtle but significant effects. Full details of the post hoc testing are shown in online supplementary table S3.

## Discussion

This study produced three key findings. First, task prioritisation is perceived to be the most important non-technical skill by medics throughout their training. Second, this study suggests a significant discrepancy between the expectations of and the reality experienced during OoH work. Third, the importance placed on specific non-technical skills changes with increasing seniority.

Our study has strengths in its investigation of a vital but under-researched area of medical care, and it is possible to draw conclusions with practical consequences. We are reassured by the statistically significant and consistent effects even after a conservative correction for multiple testing, suggesting that the relatively small sample size was sufficient to identify a true pattern. The survey was subject to bias from both its geographical and contextual deployment (teaching hospitals), and that as purely a self-report, the study could have been influenced by responder bias.

To the best of our knowledge, no other study has specifically investigated medical trainees’ perceptions of which non-technical skills are most important. Our findings are consistent with a recent study that analysed direct measures of junior doctor activity to show that newly qualified staff took less time to complete non-urgent tasks than those who were 1 year more experienced, but longer to complete urgent tasks. This would suggest that new F1s are influenced more by location and time of task allocation than clinical priority.^11^

Communication skills are clearly important to successful medical practice and are now a key feature of medical school curricula. It is notable, however, that communicating with colleagues was rated as more important than communication with patients in our study. This observation would be consistent with the observational studies on team-working largely undertaken in relation to surgery.^18^ ^19^ ^23^ Communication skills are a key part of most UK medical courses, but they tend to focus solely on patients with specific problems or those who have an emotional barrier to communication, at the expense of exploring communication with other staff, which has been highlighted as just as important in this study. Training in this time-pressured interspecialty communication through simulation or role play has great scope to improve the safety and timeliness of medical care.^24^ This approach could also benefit other healthcare professionals: a recent study revealed that nurses call doctors around half as often as local guidelines mandate they should.^25^

This study highlights a significant discrepancy between the expectations of and preparedness for the reality experienced during OoH work. The increase in student numbers and changes to teaching and working patterns seem to have introduced an ‘apprenticeship gap’: students are not fully aware of the need for non-technical skills and take time to acquire them once they begin practising. Although medical school curricula are under constant revision to ensure that technical information is correct, we suggest that this process may have been less assiduous for non-technical aspects and agree that there is too great a burden on the shadowing period.^26^ Guidance for students from professional bodies may reinforce this disconnect as they focus on sleep management,^27^ which in our survey is the least important non-technical skill of first year doctors.

This study, combined with others looking at data directly measuring junior doctor activity,^11^ suggests that teaching task prioritisation should be a greater priority for medical schools. Future research opportunities lie in investigating the optimal manner in which to teach non-technical skills, particularly in the use of simulation and serious video games. Multicentre trials conducted with robust methodology will be required to establish if such interventions have beneficial effects on workforce efficiency and on clinical outcomes such as length of stay and reported adverse incidents.
